# The possibility of identifying brain hemorrhage in putrefied bodies with PMCT

**DOI:** 10.1007/s12024-020-00283-8

**Published:** 2020-08-25

**Authors:** Carlo Tappero, Michael J. Thali, Wolf Schweitzer

**Affiliations:** 1 Department of Forensic Medicine and Imaging, Institute of Forensic Medicine, University of Zurich, 8057 Zurich, Switzerland; 2 Radiology Department, HFR Freiburg – Kantonsspital, Chemin des Pensionnats 2-6, 1752, Villars-sur-Glâne, Switzerland

**Keywords:** Postmortem imaging, PMCT, PMMR, Intracranial hemorrhage, Putrefied bodies, Liquefied brain

## Abstract

This paper aims to demonstrate that post-mortem CT (PMCT) can locate intracranial hemorrhages, even in decomposed cases. This is of relevance in that post-mortem decomposition is particularly damaging to the brain tissue’s consistency, resulting in great difficulties to reliably diagnose and locate intracranial hemorrhages. We searched our case database of the last 11 years to find cases with decomposition of the body, where PMCT and an autopsy had been performed. We identified eleven cases according to these criteria. Postmortem interval ranged from 2 days to 2 weeks, and post-mortem radiological alteration index (RAI) was at or above 49. Eight out of eleven cases showed an intraparenchymal hemorrhage whereas the hemorrhage was extra-axial in the remaining three cases. Autopsy validated the presence of intracranial hemorrhage in all eleven cases, but location could not be confirmed due to liquid state of the brain. PMCT identified and localized intracranial hemorrhages in decomposed bodies, and in all of these cases, autopsy validated their presence. The actual cause of the hemorrhage (e.g. tumor, metastasis, vascular malformation, hypertensive hemorrhage) remained obscure. From this case series, it can be concluded that PMCT may add relevant information pertaining to localization of intracranial hemorrhages in decomposed bodies.

## Introduction

Detection and specification of intracranial hemorrhages are an important part of an examination, before establishing a cause of death [1]. Discriminating intraparenchymal hemorrhages [2] from extra-axial hemorrhages [3, 4] appears to be relevant with regard to manner of death, particularly in that identifying a mass hemorrhage or subdural bleeding has different consequences for the way a case is subsequently undertaken. So, identifying a natural cause of death in an initially suspicious or unclear situation may defuse the pressure on the investigating authorities and allow them to release the body and the secured premises [5, 6].

Typically the brain decomposes after death by becoming liquefied, which can occur as soon as 2–3 days after death [7]. Putrefaction thus reduces the examiner’s capability to provide sufficient diagnostic distinction between fall or blow related hemorrhages when compared to the consequences of a hypertensive mass hemorrhage. In decomposed bodies, autopsy may still allow the identification of reddish discoloration of the liquefied brain, but localization of this discoloration may be difficult or impossible. Performing postmortem imaging prior to opening the skull has been found to be helpful, particularly in these instances [8].

As forensic imaging may be performed by clinical radiologists who have not had experience with images in which the brain anatomy is completely changed, such as changes like the loss of differentiation between gray and white matter, sulci and gyri or ventricles that may not be identifiable, or the presence of gas inside the skull or in the soft tissue, or sedimentation of the structure, any degree of post mortem decomposition has the tendency to appear new and unusual.

In bodies with no signs of putrefaction the overall specificity for intra- and extra-axial brain findings, compared to autopsy, was reported as 94% for both PMCT (post mortem computed tomography) and PMMR (post mortem magnetic resonance) [9]. PMMR can identify brain morphology and pathological findings in softened or liquefied brains, even when they are not detectable in PMCT scan or at autopsy [10]. In recent case reports, PMCT identified an intraparenchymal brain hemorrhage in decomposed bodies three [11] and four days [12] after death. To be detected, the hemorrhage has to exceed a size of about 5 mm.

This study presents a series of eleven putrefied bodies with an estimated post mortem interval between two days and two weeks. In all cases, routine PMCT performed before the autopsy had shown an intracranial hemorrhage.

## Methods and material

We conducted a review of all PMCT-scans performed at our institute in the years 2007 through 2018 to first extract a case series with the diagnosis of an intracranial hemorrhage where an autopsy had been performed. In this group (*n* = 14), we selected only cases with evident signs of decomposition, characterized specifically by gas collection in the brain and in soft tissue with a post-mortem radiological alteration index (RAI) [13] at or in excess of 49 (Table 1). The post-mortem RAI quantifies the presence of gas in PMCT to categorize cases of post-mortem decomposition. Thereby, the amount of gas is scored across various parts of the body as shown in PMCT (details of the method described in [13]). Eleven cases met all of these criteria. Time of death estimates ranged from two days to two weeks before PMCT.

**Table 1:** Ra index. The cases from 1 to 11 were those analyzed in this study

Case #	Heart cavities	Liver Parenchyma & vessels	Left innominate vein	Abdominal aorta	Kidney parenchyma	Vertebra L3	Subcutaneous pectoral tissues	Index
1	17	20	15	8	7	10	8	85
2	17	20	15	8	7	25	8	100
3	8	5	15	8	0	5	8	49
4	17	20	15	8	7	5	8	80
5	17	20	15	8	7	10	8	85
6	17	20	15	8	7	25	8	100
7	17	20	15	8	7	25	8	100
8	17	20	15	8	7	25	8	100
9	17	20	15	8	7	25	8	100
10	17	20	15	8	7	10	8	85
11	17	20	15	8	7	25	8	100
12	1	5	5	8	0	5	8	32
13	8	1	5	8	0	5	8	35
14	8	5	5	8	7	5	8	46

Autopsy removal of the brain was performed according to the Flechsig method. This method is a forensic pathology standard, where the skull is cut with a saw line, but not initially opened entirely, and the brain then is cut through that saw line, allowing an immediate view of the brain cross section, without the delay usually caused by removing the skull-cap first. This technique is described in more detail by Cattell [14].

The PMCT was performed on a 2 × 128-row SOMATOM Definition Flash CT manufactured by Siemens Healthcare GmbH, Erlangen, Germany. We used a tube voltage of 120 kV, an automatically modulated tube current (mAs) with a maximum of 400mAs for the body and 800mAs for the brain; acquisition parameters of 128 × 0.6 mm and a pitch factor 0.35. The acquired images were reconstructed with a slice thickness of 0.6 mm with an increment of 0.4 mm, and with a slice thickness of 4 mm with an increment of 3 mm, both with a Medium Smooth+ Kernel (Siemens H31s). Data were analyzed with syngo.via (Siemens Healthcare GmbH, Erlangen, Germany) version 04.01.0000.0001. Regions of interests were placed manually for density determination.

## Results

In the eleven cases that were investigated, the post-mortem interval ranged from an estimated two days to about two weeks.

In all eleven cases, the PMCT showed hyperdense pathology, typical for intracranial hemorrhage, of at least 5 mm extent. Identification of hemorrhagic areas in the liquefied brain was possible in all eleven cases. The reason was the higher density of blood relative to brain tissue: the PMCT density of the brain was 42.01 ± 3.75 Hounsfield units (HU), whereas hemorrhages yielded a density measurement of 74.67 ± 5.43 HU [12].

The possibility to visually differentiate brain structures and ventricles on PMCT decreased with postmortem interval [10] as listed in Table 2. The gray and white matter junction and the basal ganglia could not be identified in any of the cases. Also, a number of anatomical brain substructures could not be identified. Only the ventricles remained identifiable up to 14 days after death.

**Table 2:** Identification of brain structures

ID	PMI (days)	Gray and white matter junction	Ventricular system	Basal ganglia	Brain stem
1	3–7	no	yes	no	no (artifact)
2	7–14	no	partially	no	no
3	3–5	no	yes	no	no
4	2–7	no	partially	no	no
5	1.5–3	no	yes	no	no
6	7–14	no	no	no	no
7	14–30	no	no	no	no
8	3–5	no	yes	no	no
9	3–8	no	yes	no	no
10	2–7	no	yes	no	no
11	5–10	no	no	no	no

**Fig. 1:**
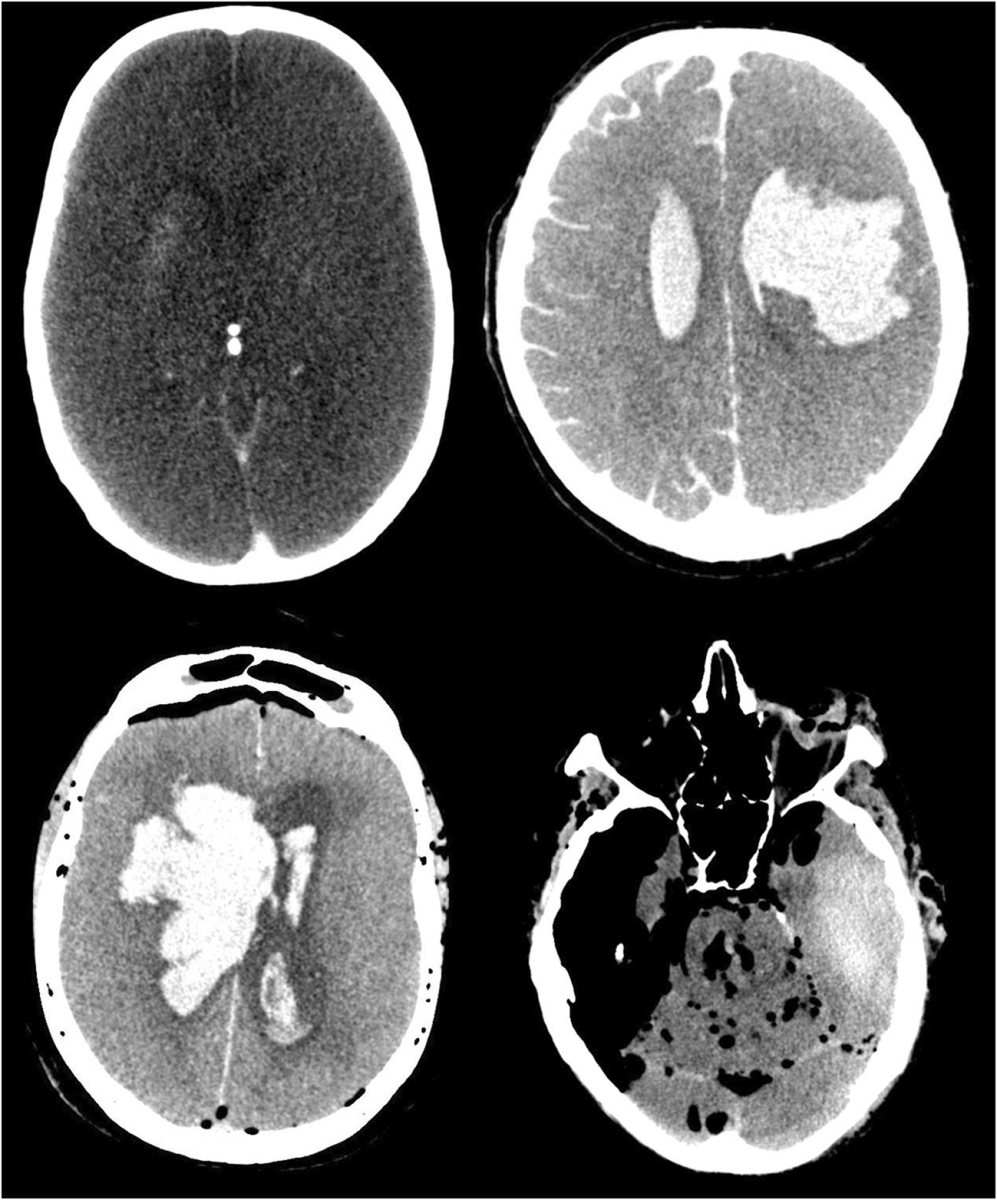
Intraparenchymal bleeding

In 72.7% (8/11) of the cases, an intraparenchymal hemorrhage was found. In all cases, the hemorrhages were regarded as potentially fatal (Fig. 1), due to both localization and extent. None of these cases showed skull fractures or other injuries of potentially lethal significance.

**Fig. 2:**
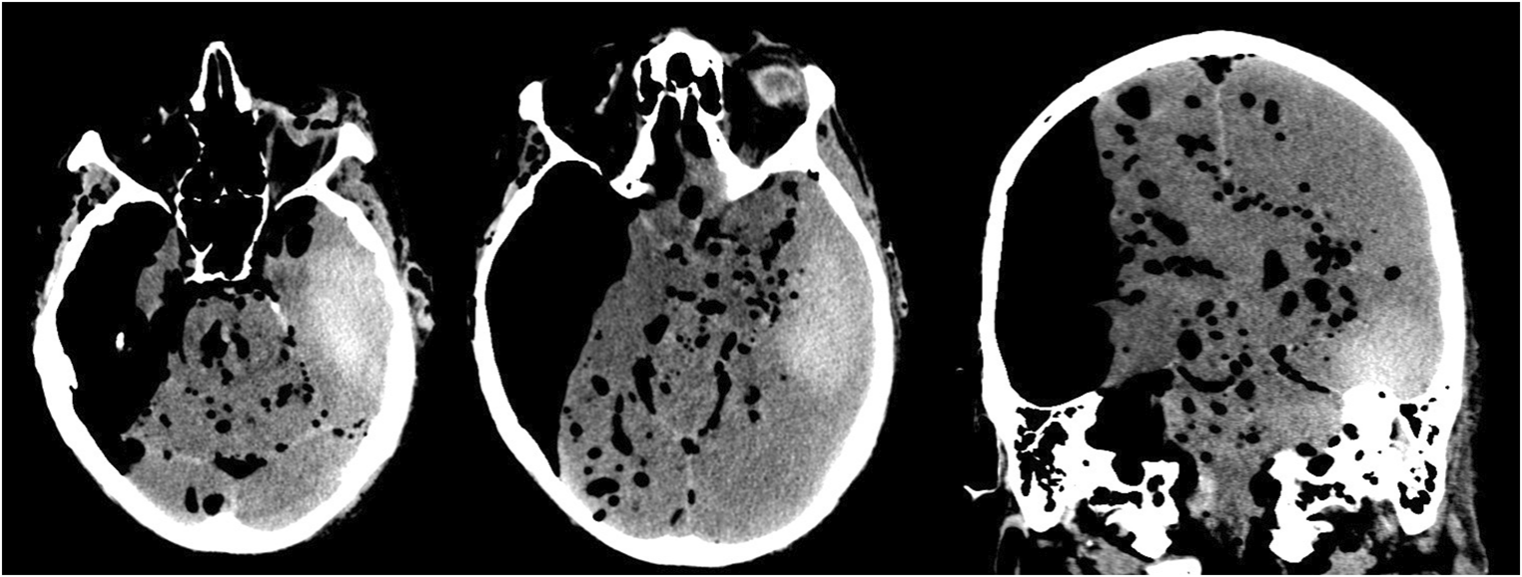
Temporal bleeding shown by CT

In seven of the eight cases (87.5%) with intraparenchymal bleeding, the hemorrhage was within the regions of the basal ganglia, cerebellum or occipital lobe. In one case, the hemorrhage was in the left temporal lobe. That is an unusual site for hypertensive hemorrhage or embolic infarction (Fig. 2) and for that reason, it could be a sign of a different underlying pathology. The hemorrhage exhibited an intraventricular invasion in six cases.

**Fig. 3:**
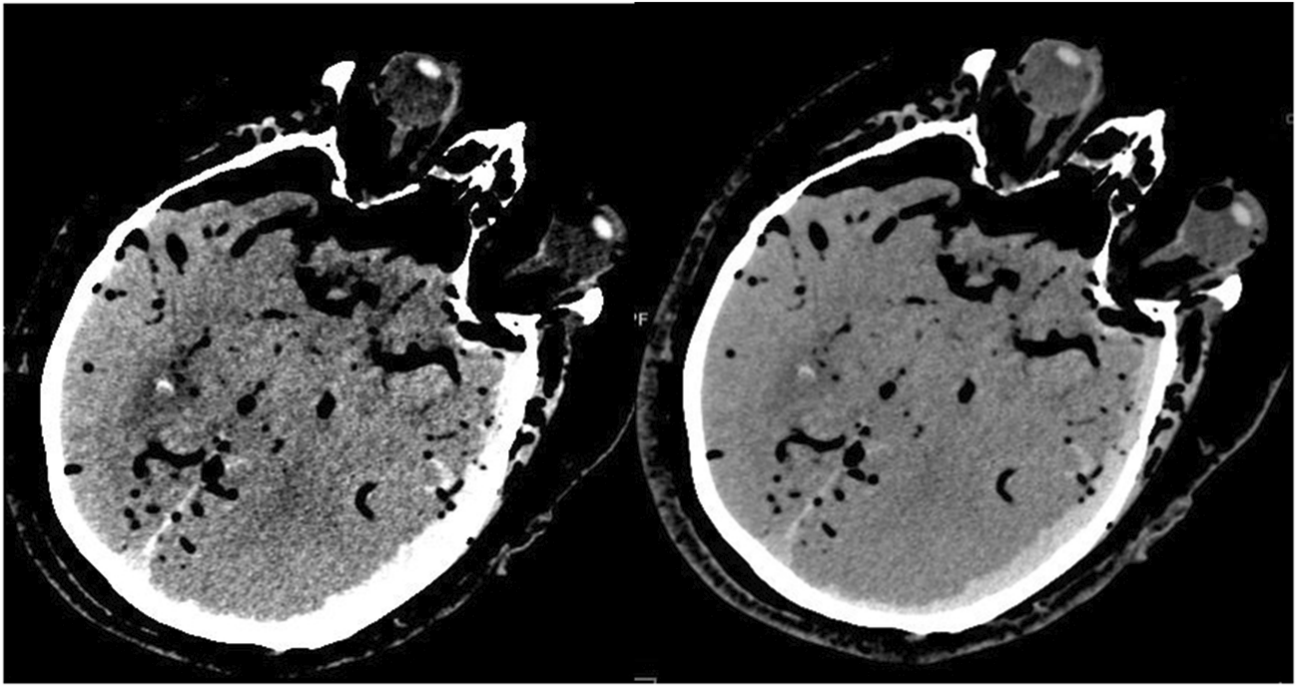
Subdural hematoma before (left) and after (right) windowing

In three cases (3/11), the bleeding was extra-axial. The hemorrhages in these cases were also regarded as potentially fatal due to their extent. In one case, there was a subdural hematoma and a subarachnoid hemorrhage. These appeared to be in conjunction with a skull burst fracture on the left side, through the temporal and parietal bones. In another case, classification of the hemorrhage was difficult because of the distribution of the blood near the left parietal bone, which could only be visualized by manually adjusting the window level (Fig. 3); there was a fracture located through the left occipital bone. The last case had subarachnoid bleeding without any skull fractures.

In all eleven cases, autopsy confirmed an intracranial hemorrhage. In all cases, the brain tissue appeared to exhibit a higher viscosity than blood, in other words, a higher mechanical consistency.

**Fig. 4:**
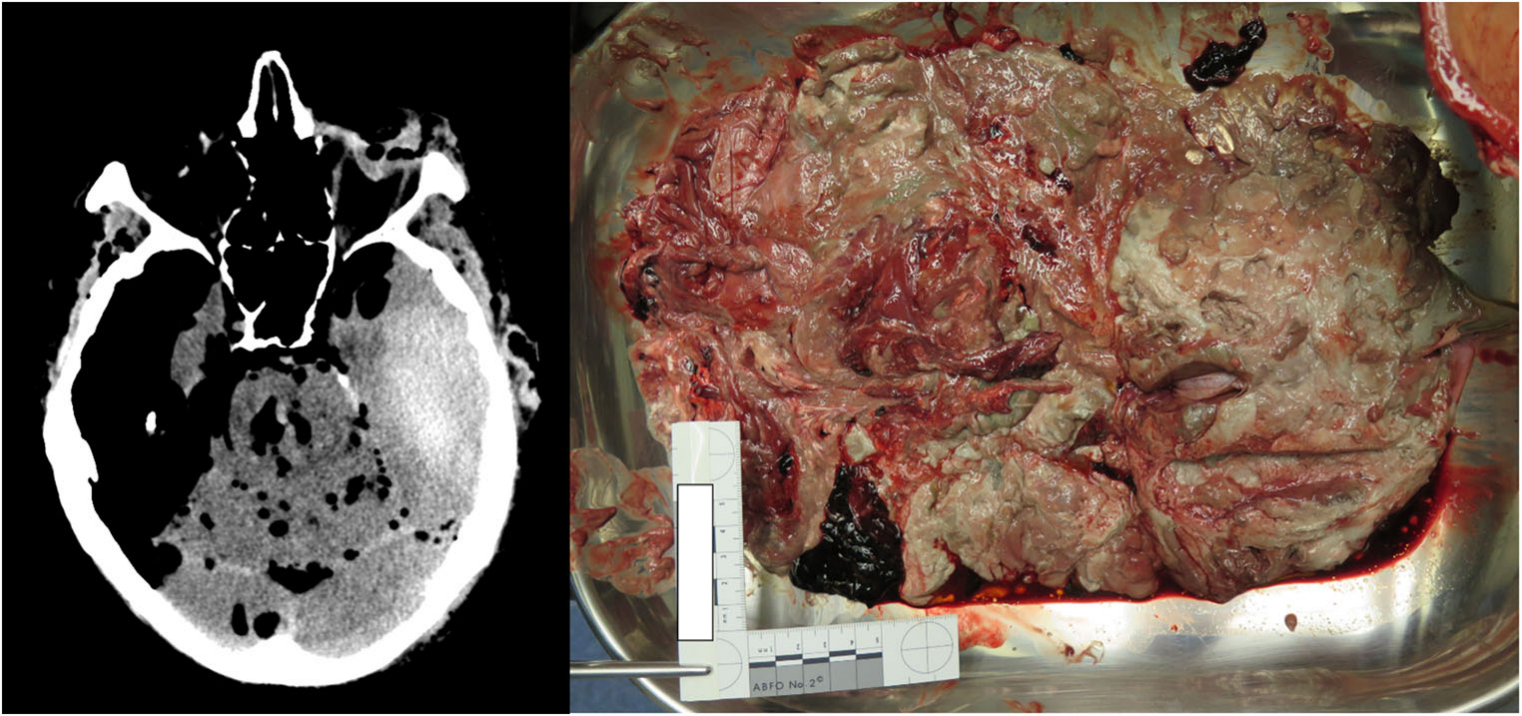
Comparison of CT results with those of autopsy, in which the brain poured out of the skull

After the opening of the skull, the brains were cut using the standard Flechsig transverse skull cut. Immediately, the liquefied brain drained into the metal tray, mixing with any coagulated or liquefied blood already present or also draining into the tray (Fig.4). This was a particular diagnostic problem in cases with an intraparenchymal brain hemorrhage. As opposed to PMCT, identifying the location of blood at autopsy was often not possible. In all of these cases, liquefied blood appeared to be more fluid (and thus, less viscous or less consistent) than the liquefied brain. Diagnosing the extent and location was easier at autopsy for extra-axial hemorrhages: there, both methods (PMCT, autopsy) clearly identified the location of the hemorrhage.

## Discussion

Our study showed that PMCT reliably identified intracranial hemorrhages in eleven cases with a post-mortem interval of two days to two weeks and a relevant degree of postmortem decomposition (RAI > 49). PMCT is powerful enough to identify intracerebral or extra-axial hemorrhages and localize the hemorrhage, and in cases of intraventricular invasion, even that anatomical aspect may still be identifiable.

Liquefaction of the brain may occur as a regular part of post-mortem decomposition. The associated loss of consistency or viscosity of the brain may render autopsy diagnosis of intraparenchymal cerebral hemorrhage difficult and localization impossible. As far as technical feasibility is concerned, autopsy confirmed the intracranial hemorrhage in all eleven cases of our series. However, in cases with a particularly high degree of liquefaction, autopsy diagnosis was possible only within a few seconds of observing the macroscopic finding, right after the Flechsig transverse cut was made.

According to the PMCT findings, intracerebral hemorrhages appeared to remain located within the brain tissues, up to the longest post-mortem interval included in this study (two weeks after death), despite the liquefaction of both brain and clotted blood. The question is, why do intraparenchymal hemorrhages appear to stay where they must have initially appeared? In our view, this may be related to our observation, that despite the presence of marked post-mortem decomposition, the brain maintained a higher relative viscosity than that of liquefied and previously possibly clotted blood.

Intraparenchymal brain hemorrhages appeared to be exceedingly difficult to localize correctly at autopsy. Conversely, extra-axial hemorrhages were easier to identify and localize at autopsy despite decomposition. However, they are difficult to reliably interpret reconstructively. From a neuroradiological experience, subdural or subarachnoid hemorrhages are known to undergo repositioning or relocation either antemortem [15] when there is already a lesion of the dura because of previous trauma, or, possibly, postmortem [9]. This is even more so the case with increasing liquefaction of the brain over time due to postmortem decomposition.

Recent studies have demonstrated that it is still possible, even in a decomposed and liquefied brain, to identify the different brain structures (gray and white matter and ventricles) with the help of PMMR [10]. By contrast, autopsy or a PMCTA (postmortem CT angiography) may actually identify a vascular source of the hemorrhage, however much this was not a focus of this study.

From our series, PMCT of intracranial hemorrhage in putrefied bodies has the following limitations:

1. it may be able to identify the hemorrhage, but the actual cause (tumor, metastasis, vascular malformation, hypertensive hemorrhage) typically remains obscure.
2. a subdural or subarachnoid hemorrhage may shift, relocate or reposition over time.
3. for an intracranial hemorrhage to be detected, the hemorrhage may have to exceed a size of approximately 5 mm.

## Conclusion

PMCT allows the identification of fatal brain hemorrhages even in cases with putrefaction or decomposition, where autopsy, due to liquefaction of the brain tissues, is hard to perform. Even though the actual cause of the hemorrhage may remain obscure. In addition, due to a long post-mortem interval, shifting of the blood may obscure its original location particularly in extra-axial bleedings.

## Key points

1. Post mortem CT is a useful tool to analyze putrefied bodies where tissues exhibit major alterations compared to a recently deceased person.
2. Post mortem CT can identify intracranial hemorrhage even when liquefied.
3. Even in liquefied brain, it is possible to identify the location of the bleeding with PMCT. This is lost after opening the skull.
